# Supplementary material for: From sequence to enzyme mechanism using multi-label machine learning
Source: BMC Bioinformatics. 2014 May 19;15:150. doi: 10.1186/1471-2105-15-150 (PMC4229970; doi:10.1186/1471-2105-15-150)
Supplement: Additional file 2 — Java code of ml2db. Additional file ml2db_code.tar.gz contains the Java source code to run the multi-label machine learning experiments and save the results to database. The code’s Javadoc is included. [file 1471-2105-15-150-S2.zip › additional file 2/ml2db/ml2db/doc/mulan/classifier/meta/SubsetLearner.html]

SubsetLearner


JavaScript is disabled on your browser.


- Overview
- Package
- Class
- Use
- Tree
- Deprecated
- Index
- Help

- Prev Class
- Next Class

- Frames
- No Frames

- All Classes

- Summary:
- Nested |
- Field |
- Constr |
- Method

- Detail:
- Field |
- Constr |
- Method


mulan.classifier.meta

## Class SubsetLearner

- java.lang.Object
- - mulan.classifier.MultiLabelLearnerBase
  - - mulan.classifier.meta.MultiLabelMetaLearner
    - - mulan.classifier.meta.SubsetLearner

- All Implemented Interfaces:
  :   java.io.Serializable, MultiLabelLearner, weka.core.TechnicalInformationHandler

  ---

    

  ```
  public class SubsetLearner
  extends MultiLabelMetaLearner
  ```

  A class for learning a classifier according to disjoint label subsets: a multi-label learner (the Label Powerset by default) is applied to subsets with multiple labels and a single-label learner is applied to single label subsets. The final classification prediction is determined by combining labels predicted by all the learned models. Note: the class is not multi-thread safe. <br> <br> There is a mechanism for caching and reusing learned classification models. The caching mechanism is controlled by {@link #useCache} parameter.

  Version:
  :   30.11.2010

  Author:
  :   Lena Chekina (lenat@bgu.ac.il), Vasiloudis Theodoros

  See Also:
  :   Serialized Form

- - ### Constructor Summary

    Constructors

    Constructor and Description

    `SubsetLearner()`

    Default constructor

    `SubsetLearner(int[][] labelsSubsets,
    weka.classifiers.Classifier singleLabelClassifier)`

    Initialize the SubsetLearner with labels subsets partitioning and single
    label learner.

    `SubsetLearner(int[][] labelsSubsets,
    MultiLabelLearner multiLabelLearner,
    weka.classifiers.Classifier singleLabelClassifier)`

    Initialize the SubsetLearner with labels set partitioning, multilabel and
    single label learners.

    `SubsetLearner(LabelClustering clusteringMethod,
    MultiLabelLearner multiLabelLearner,
    weka.classifiers.Classifier singleLabelClassifier)`

    Initialize the SubsetLearner with a label clustering method, multilabel
    and single label learners.
  - ### Method Summary

    Methods

    | Modifier and Type | Method and Description |
    | --- | --- |
    | `java.lang.String` | `getModel()` Returns a string representation of the model |
    | `weka.core.TechnicalInformation` | `getTechnicalInformation()` Returns an instance of a TechnicalInformation object, containing detailed information about the technical background of this class, e.g., paper reference or book this class is based on. |
    | `java.lang.String` | `globalInfo()` Returns a string describing the multi-label learner. |
    | `MultiLabelOutput` | `makePredictionInternal(weka.core.Instance instance)` We make a prediction using a different method depending on whether the split has one or more labels |
    | `void` | `resetRandomSeed(java.lang.Object model)` Invokes the setSeed(1) or setRandomSeed(1) method of the supplied object's Class, if such method exist. |
    | `void` | `resetSubsets(int[][] labelsSubsets)` Reset the label set partitioning. |
    | `void` | `setSeed()` Set random seed of all internal Learners to 1. |
    | `void` | `setUseCache(boolean useCache)` Sets whether cache mechanism will be used |

    - ### Methods inherited from class mulan.classifier.meta.MultiLabelMetaLearner

      `getBaseLearner`
    - ### Methods inherited from class mulan.classifier.MultiLabelLearnerBase

      `build, getDebug, isUpdatable, makeCopy, makePrediction, setDebug`
    - ### Methods inherited from class java.lang.Object

      `equals, getClass, hashCode, notify, notifyAll, toString, wait, wait, wait`

- - ### Constructor Detail


    - #### SubsetLearner

      ```
      public SubsetLearner()
      ```

      Default constructor


    - #### SubsetLearner

      ```
      public SubsetLearner(int[][] labelsSubsets,
                   weka.classifiers.Classifier singleLabelClassifier)
      ```

      Initialize the SubsetLearner with labels subsets partitioning and single
      label learner.
      `LabelPowerset` method initialized
      with the specified single label learner.will be used as multilabel
      learner.

      Parameters:
      :   `labelsSubsets` - subsets of dependent labels
      :   `singleLabelClassifier` - method used for single label classification


    - #### SubsetLearner

      ```
      public SubsetLearner(int[][] labelsSubsets,
                   MultiLabelLearner multiLabelLearner,
                   weka.classifiers.Classifier singleLabelClassifier)
      ```

      Initialize the SubsetLearner with labels set partitioning, multilabel and
      single label learners.

      Parameters:
      :   `labelsSubsets` - subsets of dependent labels
      :   `multiLabelLearner` - method used for multilabel classification
      :   `singleLabelClassifier` - method used for single label classification


    - #### SubsetLearner

      ```
      public SubsetLearner(LabelClustering clusteringMethod,
                   MultiLabelLearner multiLabelLearner,
                   weka.classifiers.Classifier singleLabelClassifier)
      ```

      Initialize the SubsetLearner with a label clustering method, multilabel
      and single label learners.

      Parameters:
      :   `clusteringMethod` -
      :   `multiLabelLearner` - method used for multilabel classification
      :   `singleLabelClassifier` - method used for single label classification
  - ### Method Detail


    - #### resetSubsets

      ```
      public void resetSubsets(int[][] labelsSubsets)
      ```

      Reset the label set partitioning.

      Parameters:
      :   `labelsSubsets` - - new label set partitioning


    - #### resetRandomSeed

      ```
      public void resetRandomSeed(java.lang.Object model)
      ```

      Invokes the setSeed(1) or setRandomSeed(1) method of the supplied
      object's Class, if such method exist.

      Parameters:
      :   `model` - which random seed should be reset.


    - #### setSeed

      ```
      public void setSeed()
      ```

      Set random seed of all internal Learners to 1.


    - #### makePredictionInternal

      ```
      public MultiLabelOutput makePredictionInternal(weka.core.Instance instance)
                                              throws java.lang.Exception
      ```

      We make a prediction using a different method depending on whether the
      split has one or more labels

      Parameters:
      :   `instance` - the instance for classification prediction

      Returns:
      :   the `MultiLabelOutput` classification
          prediction for the instance

      Throws:
      :   `java.lang.Exception`
      :   `InvalidDataException` - if specified instance data is invalid and can not be processed by the learner


    - #### setUseCache

      ```
      public void setUseCache(boolean useCache)
      ```

      Sets whether cache mechanism will be used

      Parameters:
      :   `useCache` - whether cache mechanism will be used


    - #### getTechnicalInformation

      ```
      public weka.core.TechnicalInformation getTechnicalInformation()
      ```

      **Description copied from class: `MultiLabelLearnerBase`**

      Returns an instance of a TechnicalInformation object, containing detailed
      information about the technical background of this class, e.g., paper
      reference or book this class is based on.

      **Specified by:**
      :   `getTechnicalInformation` in interface `weka.core.TechnicalInformationHandler`

      **Specified by:**
      :   `getTechnicalInformation` in class `MultiLabelLearnerBase`

      Returns:
      :   the technical information about this class


    - #### getModel

      ```
      public java.lang.String getModel()
      ```

      Returns a string representation of the model

      Returns:
      :   a string representation of the model


    - #### globalInfo

      ```
      public java.lang.String globalInfo()
      ```

      **Description copied from class: `MultiLabelLearnerBase`**

      Returns a string describing the multi-label learner.

      **Specified by:**
      :   `globalInfo` in class `MultiLabelLearnerBase`

      Returns:
      :   a description suitable for displaying in a future gui


- Overview
- Package
- Class
- Use
- Tree
- Deprecated
- Index
- Help

- Prev Class
- Next Class

- Frames
- No Frames

- All Classes

- Summary:
- Nested |
- Field |
- Constr |
- Method

- Detail:
- Field |
- Constr |
- Method
